# Evidence for incentive-based strategies to promote breastfeeding: a systematic literature review of randomised controlled trials

**DOI:** 10.1186/s12884-025-08405-2

**Published:** 2025-11-19

**Authors:** Elizabeth M. Camacho, Kym A. Reyes

**Affiliations:** 1 Institute of Population Health, University of Liverpool, Liverpool, UK; 2 School of Medical Sciences, University of Liverpool, Liverpool, UK

**Keywords:** Breastfeeding, Milk, Incentive, Reward, Systematic review

## Abstract

**Background:**

There is some evidence that incentive-based strategies effectively encourage smoking cessation in the perinatal period. Incentives could be part of policies aiming to increase breastfeeding rates. This systematic review aimed to summarise current evidence to guide researchers and policymakers towards potentially effective incentive-based strategies for increasing breastfeeding.

**Methods:**

Searches of electronic literature databases were conducted (MEDLINE, Scopus, Embase, CINAHL, and PsycInfo) for evaluations of incentive-based strategies to promote breastfeeding published up to August 2024. Identified studies were screened against pre-specified inclusion criteria: studies focusing on promoting or sustaining breastfeeding; an incentive intervention given to mothers or households; a comparator of standard or usual care or an alternative non-incentive intervention; random allocation to treatment group; evaluation of at least one quantitative outcome measure related to breastfeeding. The following were excluded: literature reviews; conference abstracts; protocol papers; animal studies. Key study information was extracted from included records and they were critically appraised using a published checklist (CASP RCT). The results were presented and synthesised narratively. The review protocol was published on the PROSPERO literature review register.

**Results:**

Database searches identified 64 non-duplicate records, and 2 additional records were identified through citation searching of previously published reviews. There were 7 records (from 6 studies) included in the review. Four studies included a total of 260 participants, and the other two studies included a total of 3418 households. Most studies were either conducted in low-income countries (3 of 6) or with low-income mothers in the United States (2 of 6). Some studies reported positive effects of incentives on breastfeeding intention, initiation, prevalence, and duration of exclusive breastfeeding, although others reported neutral findings. Incentives and study designs were heterogeneous, the studies were generally small, and results may not be generalisable to other settings/population groups.

**Conclusions:**

There is some evidence that incentives may improve breastfeeding outcomes, although the quantity and quality of current evidence are both low. Future studies should ensure that sample sizes are sufficiently large and that core breastfeeding outcomes are defined and collected.

**Supplementary Information:**

The online version contains supplementary material available at 10.1186/s12884-025-08405-2.

## Background

The World Health Organisation (WHO) recommend initiation of breastfeeding within 1 h of birth and exclusive breastfeeding for the first 6 months of life [1]. However, globally only 44% of infants aged 0–6 months were exclusively breastfed between 2015 and 2020. There is variation in exclusive breastfeeding rates globally with lower rates in Eastern Mediterranean and European regions and upper middle-income countries (versus South-East Asia/Western Pacific and lower middle-income countries, respectively) [2]. The costs associated with potentially avoidable child and maternal morbidity (e.g. preventable infections) and deaths, and cognitive losses from not breastfeeding are estimated to represent between 0.49% and 0.70% of global gross national income [3, 4]. Optimal breastfeeding could save the lives of an estimated 823,000 children aged under 5 annually [5]. Increasing the level of breastfeeding is a flagship policy for health promotion organisations globally.

Many existing interventions to promote breastfeeding focus on education and support (either from a professional or peer-supporter) [6–8] and these interventions can help to address individual and institutional barriers to breastfeeding [9, 10]. However, motivation can be a key personal barrier to breastfeeding which these interventions are less likely to address. The personal, emotional, and social barriers to breastfeeding (and other positive health behaviours) can be conceptualised as a ‘metaphorical ladder’ [11]. Giving people incentives, like money or vouchers, is a way of providing ‘rungs’ to help or motivate them to climb the ladder. A previous literature review identified some evidence that financial incentives can effectively encourage healthy behaviour change in the general population [12]. A recent Cochrane review synthesised evidence from around the globe for incentives for smoking cessation in the general population [13]. The review also considered a subgroup of 13 studies which included 3942 pregnant women. These studies produced high-certainty evidence that incentive schemes improve smoking cessation in the perinatal period. However, less is known about the use of incentive-based strategies to promote breastfeeding. A literature review of the use of incentives to change a range of maternal health behaviours (including breastfeeding) among women in the United States reported that there was some evidence to suggest that financial incentives were effective [14]. A review of studies published over a decade ago (up to May 2012) reported that due to a lack of high-quality studies and study heterogeneity there was no clear conclusion on the effectiveness of incentives [15]. It is important to keep revisiting this topic to bring renewed awareness and to identify and highlight any opportunities for learning from advances in knowledge.

This review sought to produce an up-to-date synthesis and critical appraisal of current knowledge about incentive-based strategies to promote breastfeeding with the aim of informing the research agenda in this area.

## Methods

We conducted a systematic search of literature published in indexed journals which reported the results of randomised evaluations of incentive-based strategy to support and/or promote breastfeeding. The primary research objective was to identify specific incentive-based strategies that showed the greatest promise of effectiveness in supporting/promoting breastfeeding. A second objective was to identify key knowledge gaps to inform recommendations for future research.

### Search strategy

A broad search strategy to maximise the likelihood of identifying all relevant literature was employed. This applied to search terms, interventions (as long as there was some incentive component), and population (not limited to specific sub-groups). The review question in PICOS format is shown in Table 1.

**Table 1:** PICOS summary of the review question

Population	mothers or households in the perinatal period (during pregnancy or in the first year postnatally)
Intervention	any type of incentive intervention
Comparator	standard or usual care, or an alternative intervention that does not include an incentive component
Outcome	evaluation of at least one quantitative outcome measure related to breastfeeding
Study design	random allocation to intervention or comparator

As per the PICOS statement, the following explicit inclusion criteria were applied: (a) studies focusing on promoting or sustaining breastfeeding, (b) any type of incentive intervention given to mothers or households in the perinatal period (during pregnancy or in the first year postnatally), (c) a comparator of standard or usual care, or an alternative intervention that does not include an incentive component, (d) random allocation to intervention or comparator, and (e) evaluation of at least one quantitative outcome measure related to breastfeeding. The following explicit exclusion criteria were applied: (a) literature review, (b) conference abstract, (c) protocol paper, or (d) animal study. Studies violating these criteria did not proceed to the next stage of the review.

Electronic databases of published literature were searched: MEDLINE, Scopus, Embase, CINAHL, and PsycInfo. The searches were restricted to studies published between 1 st January 2000 and 31 st August 2024 and those published in the English language. The authors were not able to translate reliably from other languages and studies from prior to the year 2000 are likely to be less relevant for current policymaking. The search strategy included terms to identify breastfeeding, incentives, and randomised evaluations. The full search strategy is reported in Supplementary Material (Table S1).

When literature reviews were identified, the studies included in those reviews were assessed for inclusion in this review as an opportunity to capture anything relevant not identified in our searches [8, 14–17].

### Article screening and review

The lists of studies identified from the different databases were combined and then de-duplicated. The abstracts of all studies were compared against the inclusion and exclusion criteria by both authors independently. For those studies whose abstracts conformed to the criteria, both authors then separately reviewed the full texts, applying the same criteria to identify studies that would progress to the data extraction stage. At both screening stages, the authors compared their results and discussed any incongruences until agreement was reached. For each study that underwent full text review and deemed ineligible for inclusion, the primary reason for exclusion was recorded and is reported alongside the study details in Supplementary Material (Table S2).

A combined form for data extraction and critical appraisal was generated prior to screening, based on the review protocol which was registered on the PROSPERO register of literature reviews (ID, CRD42023465949). The CASP checklist for RCTs was used for critical appraisal of included publications [18]. Data extraction was conducted by one author (author 1) and was reviewed for all included studies by another author (author 2). Information extracted from studies included in the review was summarised descriptively. No quantitative synthesis was conducted.

## Results

### Study selection

**Fig. 1:**
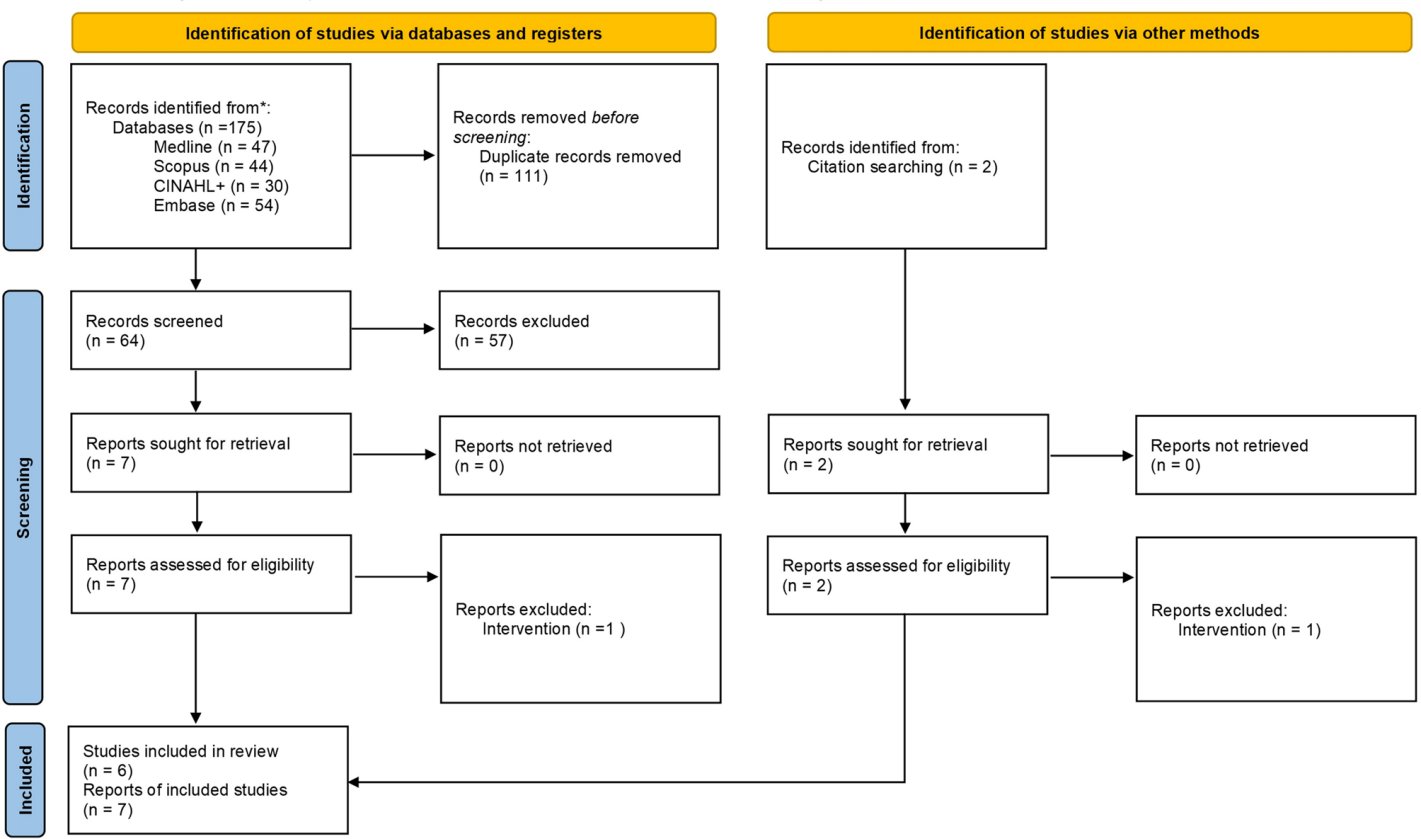
PRISMA flow diagram

The results of the searches and screening are summarised in Fig. 1. Seven publications from six studies were included in the review [19–25]. For one of the studies there were two separate publications, one reporting effectiveness [22] and another reporting cost-effectiveness results [23]. Details of the two studies which were excluded after full text review are reported in Supplementary Material. In both of these studies, participants in the control group also received an incentive [26, 27].

### Study characteristics

The study characteristics are summarised in Table 2. Half of the studies were conducted in high-income countries and half in low- or middle-income countries. Both of the studies conducted in the USA targeted low-income mothers [19, 21]. Three studies used cash transfers as the incentive intervention [20, 21, 24], one used shopping vouchers [22], and one used a maternal ‘starter kit’ [25] – all were conditional on either breastfeeding or attendance at clinics or education/training sessions. The other study used an incentive marketing activity (although details of what this entailed were not provided) [19].

**Table 2:** An overview of the studies which were included in the review (*n* = 6)

Author (year of publication(s))	Country	Population	Incentive intervention (studies reported usual or routine care as the comparator unless otherwise stated)
Finch (19)	USA	WIC participants who were English speaking, pregnant, and HIV negative	Incentive marketing in the form of a truth or myth activity in a small group format.
Kandpal (20)	Philippines	Households with estimated per capita income below the poverty line with children 0–14 years of age and/or a pregnant woman.	A combination of health grants and education grants every 2 months ranging from 500 10 1400 Philippine Pesos per household depending on the number of eligible children in the household. Grants were conditional on visits to health centres, administration of deworming medication, and attendance at family development sessions.
Washio (21)	USA	Puerto Rican women enrolled in a WIC program who had initiated breastfeeding.	The incentive amount was $20 (in cash) at the end of the first month and increased by $10 every month until the end of 6 months. The incentive was conditional of demonstration of breastfeeding.
Relton (22) Anokye (23)	UK	Mother-baby dyads living in an intervention electoral ward area.	The scheme offered shopping vouchers worth £40 (US$50) 5 times based on infant age: 2 days, 10 days, 6 to 8 weeks, 3 months, and 6 months (i.e. up to £200/US$250 in total). The incentive was conditional upon the baby receiving any breastmilk.
Kurdi (24)	Yemen	Female relatives of beneficiaries of the Social Welfare Fund (Yemen’s main social protection program) who had children under 2 years of age or were pregnant.	Monthly cash transfers of 10,000 Yemeni riyals (25% of the value of average monthly food spending) conditional on attendance at monthly nutritional training sessions led by locally recruited community health volunteers.
Rossouw (25)	South Africa	Pregnant women aged 18 and older living in a low-resource, low employment environment, not yet been enrolled for antenatal care.	A maternal starter-kit, valued at approximately $27.80 including blankets and hygiene products for the baby and mother in a box that could be used as a baby bath. Receipt of the kit was conditional on clinic attendance. There were also monthly visits from a community health worker which participant in the control group did not receive.

*WIC *Special Supplemental Nutrition Program for Women, Infants, and Children [federal grants to states for supplemental foods, health care referrals, and nutrition education for low-income pregnant, breastfeeding, and non-breastfeeding postpartum women, and to infants and children up to age 5 who are found to be at nutritional risk [28]

*HIV *human immunodeficiency virus*, USA *Unites States of America*, UK *United Kingdom

Table 3 summarises the design and samples of the included studies. Half of the studies used cluster randomisation [20, 22, 24] and the other half randomised individuals [19, 21, 25] to intervention or control. Two of the studies considered participation at the household level [20, 24], with the remainder considering individuals. Four studies included a total of 260 participants, and the other two studies included a total of 3418 households. Sample sizes ranged from 36 to 92 individuals and 1418 to 2000 households. Four of the studies described the ethnicity of participants, the study from the UK had a predominantly White sample (97.9%) [22], whereas the other three recruited predominantly or exclusively from racially minoritised groups [19, 21, 25].

**Table 3:** Summary of the design of the studies in the review and description of the study samples

First author (year of publication)	Cluster or individual randomisation	Total sample size	Sample size	Age (years)	Ethnicity	Sample size	Age (years)	Ethnicity
			Intervention group			Control group		
Finch (19)	Individual	60	30	< 16: 11% 16–18: 32% 19–24: 37% 25+: 21%	African American − 79% Hispanic − 16% White − 5%	30	< 16: 7% 16–18: 17% 19–24: 66% 25+: 10%	African American − 59% Hispanic − 38% White − 3%
Kandpal (20)	Cluster	1418 households	714 households	Not applicable	Not reported	704 households	Not applicable	Not reported
Washio (21)	Individual	36	18	24.1 (SD 4.7)	100% Puerto Rican – 33% US-born	18	23.0 (SD 4.6)	Puerto Rican – 39% US-born
Relton (22) Anokye (23)	Cluster	92	46	37.4 (SD 3.6)	97.9% White	46	36.2 (SD 3.0)	97.5% White
Kurdi (24)	Cluster	2000 households	1001 households	27.8 (SD 6.9)	Not reported	999 households	28.3 (SD 6.8)	Not reported
Rossouw (25)	Individual	72	39	27.2 (SD 5.8)	90% Black African	33	27.9 (SD 6.6)	88% Black African

### Results of syntheses

Table 4 summarises the outcome measures, results, and narrative critical appraisal of the included studies. A range of breastfeeding outcomes were considered: attitude, intention, initiation, and duration. Positive results were higher rates of initiation of breastfeeding [24], exclusive breastfeeding [19], and breastfeeding prevalence [21, 22]. Other studies showed no significant differences for initiation [20, 22] or exclusive breastfeeding [20, 22, 24]. None of the studies showed a significant difference in breastfeeding duration [19] or intention to breastfeed [25]. While there is some evidence of a positive effect of incentives on breastfeeding behaviour, there are also outcomes which indicate no effect. There are no studies suggesting a negative effect (i.e. less breastfeeding). When considering the different stages of breastfeeding (intention, initiation, continuation) it was not possible to say if incentives were more likely to be effective at any particular stage. This is because there was only a single study of breastfeeding intention (which found no effect) and there were mixed findings from the studies evaluating initiation [25]. Studies measured the continuation of breastfeeding in different ways including prevalence at a point in time [21, 22] or overall breastfeeding duration [19], with some studies reporting a positive effect and others no effect.

**Table 4:** Summary of the results of the studies included in the review

First author (year of publication)	Intervention type	Outcome measures	Results	Key conclusions	Appraisal summary
Finch (19)	Incentive marketing	BF duration (weeks) Prenatal breastfeeding attitude vs. breastfeeding activity	*Breastfeeding activity (intervention vs. control)* Exclusive: 47% vs. 17% (*p* =.025) Partial: 32% vs. 52% (*p* =.169) None: 21% vs. 31% (*p* =.447) *Any breastfeeding duration (median)* Intervention: 12 weeks Control: 6 weeks Difference: 6 weeks (*p* =.322) *Relationship between breastfeeding attitude and activity* Intervention: *p* <.001 Control: *p* =.218	The prevalence of exclusive breastfeeding (significant) and duration of any breastfeeding (non-significant) were higher in the intervention group than the control group. In the intervention group prenatal attitude to breastfeeding was significantly associated with breastfeeding behaviour, but not in the control group.	This was a small study with differential dropout between the intervention and control groups which are major weaknesses.
Kandpal (20)	Health and education grants every 2 months	Breastfeeding initiation within 24 h of birth; exclusive breastfeeding for 6 months	Program effect: *Breastfeeding initiation* 0.538 (−7.409 to 8.485) *Exclusive breastfeeding* −5.326 (−16.216 to 5.563)	The program had no effect on breastfeeding outcomes.	This was a large, randomised study, however the results may not be transferable to other settings. Also, the paper suggests that the outcome is self-reported but this was not fully clear.
Washio (21)	Monthly cash payments	Self-reported breastfeeding status at 1-month, 3-months, and 6-months postpartum	Breastfeeding prevalence (incentive vs. control): *1-month* 89% vs. 44%, *P* =.01 *3-months* 89% vs. 17%, *P* <.001 *6-months* 72% vs. 0%, *P* <.001	Significantly higher percentages of mothers in the incentive group maintained breastfeeding at each time point compared with the control group.	This was a randomised study which showed a benefit of the intervention, but was a very small sample of < 40 women, also in a low-income setting so not sufficient to change practice.
Relton (22)	Shopping vouchers 5 times over 6 months	Primary: any breastfeeding (i.e. exclusive or non-exclusive) at 6–8 weeks postpartum. Secondary: breastfeeding initiation; exclusive breastfeeding at 6–8 weeks	Any breastfeeding prevalence *Intervention* 37.9% (95% CI 35.0 to 40.8) *Control* 31.7% (95% CI 29.4 to 34.0) *Unadj. difference* 6.2% (95% CI 2.4 to 10.0; *P* =.002) *Adj. difference* 5.7% (95% CI 2.7 to 8.6; *P* <.001) Breastfeeding initiation *Intervention* 61.6% (95% CI 58.8 to 64.5) *Control* 57.6% (95% CI 54.1 to 61.0) *Unadj. difference* 4.1% (95% CI − 0.4 to 8.6; *P* =.07) *Adj. difference* 2.9% (95% CI − 0.4 to 6.2; *P* =.08). Exclusive breastfeeding prevalence *Intervention* 27.0% (95% CI 24.8% to 29.2%) *Control* 24.1% (95% CI 21.8% to 26.4%) *Unadj. difference* 2.9% (95% CI − 0.3 to 6.1; *P* =.08). *Adj. difference* 2.3% (95% CI − 0.2 to 4.8; *P* =.07).	There was a significantly higher breastfeeding prevalence in the intervention group when any breastfeeding was the outcome. The difference was not significant for breastfeeding initiation or the prevalence of exclusive breastfeeding.	Well-designed study with minimal bias. Could use the evidence to make recommendations to change practice. It would not have been possible to blind participants.
Anokye (23)		Incremental cost (set up and delivery of intervention); incremental number of babies breastfed at 6–8 weeks	*Incremental cost (intervention vs. control)* £9738 (8520 to 10957) *Incremental number of breastfed babies* 10 (6 to 14) *Incremental cost-effectiveness ratio* £974/additional breastfed baby	Costs were higher in the intervention group. Whether it is cost-effective depends on how much decision makers are willing to pay per additional breastfed baby.	
Kurdi (24)	Monthly cash payments	Self-reported practices of early initiation of breastfeeding (within 1 h of birth); exclusive breastfeeding up to 6-months	*Difference in breastfeeding initiation (intervention vs. control)* 15.6% (95% CI 0.0036 to 0.275) *Difference in exclusive breastfeeding (intervention vs. control)* 14.4% (95% CI −0.009 to 0.298)	The intervention was associated with a significantly higher prevalence of breastfeeding initiation a non-significantly higher prevalence of exclusive breastfeeding for 6 months.	This was a large, randomised study; however, the context is very specific to Yemen’s humanitarian context, and outcomes are self-reported, therefore it would be not sufficient for changing practice in other settings.
Rossouw (25)	Maternal starter-kit and monthly visits from a community health worker	Adapted infant feeding intention (IFI**) scale, measured a week after birth.	Difference in IFI score (intervention vs. control) *Unadj. difference* 1.035 (SE −0.762) *Adj. difference* 1.054 (SE −0.806)	Treatment group participants scored higher on the IFI scale compared to the control group, but this was not statistically significant.	Although it was randomised, this was a pilot study only with 72 participants therefore not sufficiently powered to change practice.
**IFI - additive index ranging between 0 (no intention to breastfed at all) to 8 (very strong intention to breastfeed exclusively for six months).					

The two studies which examined prevalence as an outcome had a total of 128 participants, and both reported a positive effect. One of these was the most robustly designed study included in the review (Relton et al. [22]) although it included almost entirely ethnic majority women. In contrast, the other study reporting a positive effect on breastfeeding prevalence included entirely ethnic minority women [21]. The incentives used in these studies were cash [21] and shopping vouchers which were given on the condition of babies being given breastmilk.

Most of the studies explored more than one breastfeeding outcome. Three of the studies which reported a positive impact on one outcome also reported no impact on a different outcome [19, 22, 24]. For example, one study reported a positive effect on breastfeeding prevalence but not exclusive breastfeeding [22]. Only one study used the outcome of breastfeeding duration (number of weeks with either exclusive or partial breastfeeding) [19]. This study reported that a larger proportion of people who received the incentive exclusively breastfed than the people who did not receive the intervention, but there was no difference in the median breastfeeding duration. It is possible that different incentives are more or less likely to have an impact on different outcomes, and it is not possible to determine from the published literature which breastfeeding outcome that incentives could be most useful in targeting.

Two of the studies provided incentives to households (rather than individuals) [20, 24] and between them considered breastfeeding initiation and prevalence of exclusive breastfeeding. One study showed a positive effect on breastfeeding initiation whereas the other one did not. Neither study showed an effect on exclusive breastfeeding, suggesting that household incentives may not be effective for this outcome. In both of these studies the incentives were conditional on attendance at health clinics rather than on breastfeeding itself. Further conclusions on household level incentives for breastfeeding are limited by the small number of studies.

Overall there is neither a single incentive type, nor outcome measure, included in a sufficient number of studies to draw a robust conclusion on the effectiveness of incentives or the features that make an incentive more likely to be effective on breastfeeding behaviours.

### Reporting biases

Table 5 presents the full critical appraisal of included studies using the CASP checklist. Due to the nature of incentive interventions, it would not have been feasible to blind participants to their group allocation, but none of the studies reported using blinded outcome assessors either. Many of the studies had small samples or were conducted in specific populations (e.g. low-income Puerto Rican women living in the United States [21]) or settings (e.g. the humanitarian context in Yemen [24]), which limits the applicability of the findings elsewhere. The study by Relton et al. was a large and sufficiently powered, however, 98% of the sample were of White ethnicity which therefore included fewer people from other ethnicities than the general population of the UK.

**Table 5:**
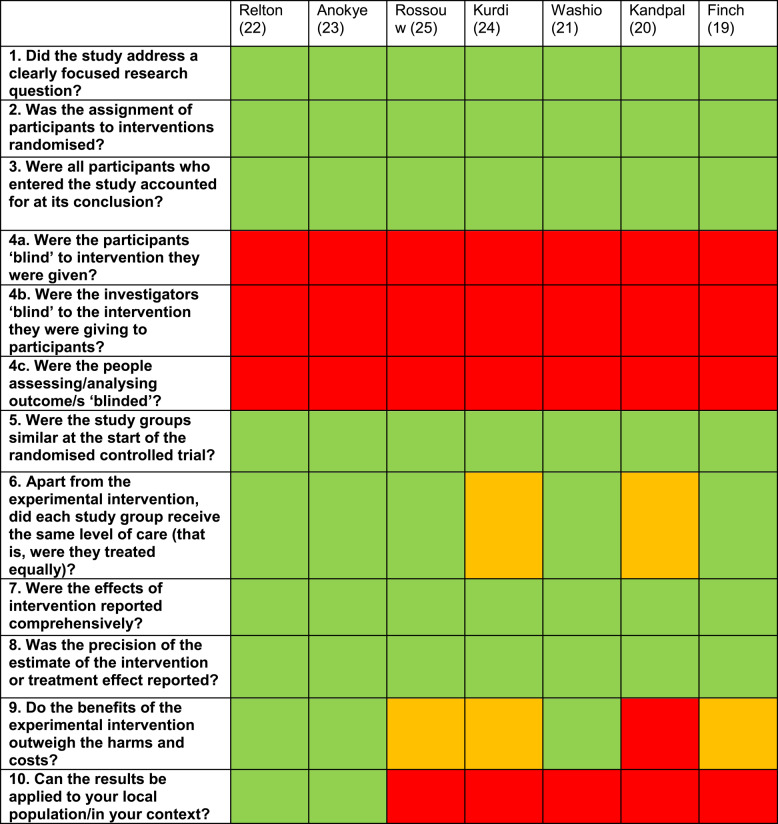
Critical appraisal using CASP checklist (18)

Green= yes; orange = unclear; red = no

Questions 4a and 4b of the checklist are not really applicable as it would not be feasible to blind people to an incentive; Question 11 of the checklist is not included as it was not applicable to incentive interventions

### Certainty of evidence

Overall the certainty of the evidence is low. This is driven by small sample sizes, heterogenous incentive interventions and outcome measures, and little to no evidence on the likely transferability of findings to other settings. The primary recommendation is the need for more robust research in the area. It is not possible to conclude strongly whether incentives are effective at improving breastfeeding outcomes.

## Discussion

The literature identified here suggests that incentive-based strategies may, in some circumstances, improve initiation of breastfeeding and rates of exclusive or any breastfeeding. However, the quality of evidence was generally low due to small sample sizes or highly specific populations which limits generalisability of findings. There were also studies which showed no significant effect of incentives on breastfeeding outcomes. Most studies were either in low-income settings or with low-income populations in high-income settings.

The findings echo those from a review of literature on incentives to promote breastfeeding that conducted searches over a decade before ours [15]. That review identified 16 studies published between 1987 and 2012 and included both randomised and non-randomised designs. Nine of the studies in that review were published before the year 2000, so the majority of evidence was from over 25 years ago. A key recommendation from that review was the need for adequately powered RCTs, which supports the focus of the current review on randomised studies. There was only one study included in that review and the current review (Finch [19]) therefore between the reviews there is a breadth of literature synthesised over almost 40 years. Overall, there is some evidence that incentives can improve breastfeeding outcomes. However, there has been limited progress in the advancement of research design in this area over time and so there is still an overwhelming need for high quality RCTs. The lack of new robust evidence in an area of such public health importance is a call to action.

Supported by a recent Cochrane review, there is consensus that incentives are effective for encouraging smoking cessation in pregnant women [13]. It may be possible to use some of the learning about how incentives for smoking cessation in this population work, to design effective incentive-based interventions for the promotion of breastfeeding, and to highlight the need to address the comparative lack of research in this area. However, there are key differences between smoking in pregnancy (a negative health behaviour) and breastfeeding (a protective health behaviour) which may have implications for the effectiveness of incentives in the different contexts.

The negative impacts of smoking in pregnancy (e.g. premature birth, stillbirth) occur in a short time frame (up to 9 months). In contrast, many of the benefits associated with breastfeeding (e.g. maternal cancers, childhood infections) happen further into the future. Concepts from behavioural economics (temporal discounting) and psychology (delayed gratification) suggest that people prefer benefits that are realised sooner and so a larger incentive may be required for breastfeeding than smoking cessation in pregnancy. One reason that smoking is addictive is that nicotine stimulates the release of dopamine which gives the smoker a feeling of reward-based pleasure [29]. Therefore incentives to stop smoking may offset some of this process. Whereas the act of breastfeeding is not inherently pleasurable, therefore an incentive would need to compensate for this. However, breastfeeding is associated with the release of oxytocin [29], which stimulates feelings of human bonding rather than reward. This might suggest that incentives for breastfeeding are most needed at the initiation stage, and that through the release of oxytocin, breastfeeding could become its own incentive for sustaining breastfeeding.

When someone stops smoking, they are directly rewarded through money saved from buying tobacco products. This is also relevant to breastfeeding, as not having to pay for formula milk also saves money. However, the human cost of breastfeeding while largely intangible is not negligible [30] and so a larger compensatory incentive may be required for breastfeeding than smoking cessation.

Like other health behaviours, barriers and facilitators to breastfeeding can act at the individual, interpersonal, community, institutional/organisational, and policy/environmental levels [9, 10]. Individual factors include motivation, desire, time, or physiological factors (e.g. mastitis). Some of the breastfeeding incentive interventions identified the current and previous literature review [15] included incentives alongside education programmes. Together these interventions may help with motivation and desire to breastfeed, but not other barriers. Support with breastfeeding from family or peers (or lack thereof) is the key interpersonal facilitator/barrier. While an individual-level incentive intervention is unlikely to address this, two of the incentives identified in this review were administered at the household level [20, 24] and an RCT identified by the earlier literature review focussed on dads as breastfeeding advocates [31] which may be more relevant to interpersonal barriers. One of the household level incentives reported a positive effect on breastfeeding but the other one did not and the study with dads showed a positive effect on breastfeeding initiation but not duration. Community level factors include the normalisation of breastfeeding and access to formal breastfeeding support. Institutional factors include resources and procedures in hospitals to support and promote breastfeeding. Policy level factors include maternity leave from employment and laws and policies for employers related to breastfeeding. Incentives given to individuals or households would not have a direct impact on barriers to breastfeeding at the broader levels (community, institutional, and policy). However, if over time more individuals are encouraged to initiate and maintain breastfeeding through appropriate incentive schemes, then breastfeeding could potentially become more normalised (community-level impact).

One of the most prolific breastfeeding support programmes is the Baby-friendly Hospital Initiative which was launched by the WHO and the United Nations Children’s Fund (UNICEF) in 1991. It aims to primarily address institutional/organisational barriers to breastfeeding in hospitals. Evidence suggests that the scheme has a positive impact on short-, medium-, and long-term breastfeeding outcomes [32]. One element of the initiative aims to address community level barriers (through the provision of support after discharge from hospital) and this appears to be key for sustaining the long-term effects [32]. There is evidence that providing breastfeeding support across a combination of settings is associated with the greatest impact on breastfeeding outcomes (compared to a single setting) [7]. An umbrella review of breastfeeding interventions in low- and middle-income countries reported that there was evidence to suggest that community-based intervention packages increase the likelihood of breastfeeding initiation, however there was insufficient evidence that interventions increased the likelihood of exclusive breastfeeding [33]. At the environment level, evidence suggests that interventions to improve workplace environments for breastfeeding can increase the duration of breastfeeding [34].

Many interventions to promote breastfeeding are targeted at the individual level, with evidence suggesting that education [6], support [6, 35], improving mothers’ breastfeeding self-efficacy [36], or a combination of strategies [8] are associated with positive breastfeeding outcomes. Like education-based interventions, incentives reinforce the message that breastfeeding is a positive health behaviour that mothers are being encouraged to do. Unlike education-based interventions which focus on the long-term benefits for mothers and babies, incentives can provide a short-term reward. In this way, incentives could increase an individual’s personal motivation to breastfeed. While there is some evidence of positive effects from different types of interventions to promote breastfeeding, globally breastfeeding rates remain below policy targets. Therefore it is important that non-traditional strategies are considered. Rewards and incentives have the potential to provide an additional strategy (i.e. alongside education, support, organisational, and societal change) to encourage individual women to breastfeed.

The individual and social acceptability of incentive-based strategies to promote breastfeeding may be a key barrier, and acceptability among key stakeholders has been previous noted as an important area for future research [37]. Qualitative research with British women suggests that women value autonomy and that receiving incentives to breastfeed may make them feel pressured [11]. Research from the United States also noted the importance of maintaining free and informed choice when using incentives to promote breastfeeding [38]. Healthcare providers noted that there could be positive and negative impacts of incentives for breastfeeding and felt that it would be important that their professional integrity was not negatively impacted by incentive schemes [39]. Acceptability among the general population has also been explored. For example, a thematic analysis of readers’ comments to online news reports about financial incentives for breastfeeding in the United Kingdom reported that those commenting viewed incentives as unacceptable [40]. However, it should be noted that people who comment on online news reports are a self-selected sample and that this may not represent widely held views. A survey with a representative sample of over one thousand British adults reported that some people agreed and some people disagreed with incentives for either smoking cessation in pregnancy or breastfeeding [41]. However, there was not a clear distinction between level of disagreement against incentives to promote breastfeeding compared with smoking cessation.

The effectiveness of incentives in changing health behaviours appears to be intrinsically linked to their acceptability. A discrete choice experiment about incentives to change health behaviours in the general population found that as the effectiveness of the incentives increased, so did their acceptability [42]. However, they also found that acceptability varied by the target behaviour, notably that incentives to promote weight loss were considered more acceptable than smoking cessation. One of the reasons people commenting on online news stories objected to incentives for breastfeeding was because they perceived that incentives are less likely to be effective than other interventions, as they do not address structural or cultural barriers to breastfeeding [40]. While there may be some validity in this argument, this would also be the case for smoking cessation, yet incentives are effective in this area [13]. This highlights the importance of addressing the ongoing evidence gap on the effectiveness of incentives for the promotion of breastfeeding, as with more robust evidence of effectiveness, acceptability may also increase.

### Limitations of the evidence

In addition to the small number of studies, the biggest limitation of the evidence overall was heterogeneity in outcome measures and incentive types. For these reasons it was not possible to conduct a quantitative synthesis of the evidence. Although there is a clear global health policy aiming for exclusive breastfeeding until babies are 6 months old [1], there is not currently a core outcome set for breastfeeding studies [43]. This means that studies measure the effectiveness of breastfeeding interventions using a range of different outcomes. Developing and reaching a consensus on an outcome set for breastfeeding is an important avenue for future research.

The most common incentive type in the studies included in our review was a cash transfer, used in three studies, the other incentives (shopping vouchers, a maternal ‘starter kit’, and incentive marketing activity alongside breastfeeding education) were each only evaluated in one study. A recent Cochrane review of interventions to support ‘healthy breastfeeding mothers of healthy term babies’ included a study which evaluated an income generating activity (making soap that could be kept or sold) [44] which the review classified as an incentive [35]. We did not include this study in our review because the authors did not refer to the intervention as an incentive or reward and during our screening process, we agreed that income generating activity is not clearly an incentive. Altogether, this heterogeneity in the incentives evaluated in randomised studies makes it difficult to guide future research. However, a discrete choice experiment found that grocery vouchers were considered to be more acceptable than cash or vouchers for luxury items [42]. A review of evidence, which examined published literature through an ethical lens, concluded that as long as incentives are carefully designed, they can be ethically justified, given the clear benefits of breastfeeding [38].

### Strengths and limitations of the review

There are a number of strengths and limitations of this review. Our searches included multiple major literature databases, which increases the likelihood that key relevant studies were identified. A protocol for the review was published in the PROSPERO database and updated during the review process. We included only randomised studies to increase the likelihood of identifying high-quality evidence from studies which were subject to less bias than observational studies. Our inclusion criteria included cost-effectiveness outcomes (although we did not search specifically for them). Cost-effectiveness evidence is important to inform decision-making, yet a systematic review of cost-effectiveness evidence for breastfeeding interventions did not identify any incentive-based strategies [45]. Only one trial-based cost-effectiveness analysis (which was published since that review) was identified in the current review, so this is a clear knowledge gap.

Despite a broad search strategy, informed by previous related literature reviews (e.g [40, 45])., some relevant studies may still not have been identified by our searches. For example, multi-component interventions which included an incentive component may have been missed due to the description of the intervention. Also, studies that gave women breast pumps were not detected by our search terms as we did not consider these as incentives when developing the search strategies. A previous literature review which included evidence from randomised and non-randomised studies noted that it was the most commonly used incentive [15]. That review reported that 2 of 3 RCTs exploring breast pump provision found that it was associated with longer duration or higher occurrence of breastfeeding. Furthermore, breast pumps were found to be the most acceptable type of incentive in a British survey [41]. It could however be argued that providing a breast pump is removing a barrier to breastfeeding, rather than incentivising or rewarding it, which may be why it appears to be effective and acceptable. Nevertheless, this may be a promising avenue for future research.

Whilst we followed systematic review best practices, including strategies to minimize bias, the search was restricted to English language studies, literature published in journals (and not grey literature), and studies published since the year 2000. Each of these criteria may have introduced bias. In particular, as noted half of the studies identified were conducted in low-income settings, so there may be more evidence from these settings which are published in non-English journals.

The studies included in this review were not restricted by study setting, however the success of incentives in encouraging breastfeeding may be different in different countries and cultures. A limitation of this review is that we have considered incentives as a broad intervention category rather than exploring which specific incentives, or how they are implemented are more likely to be effective in different contexts. This is likely to depend on the specific barriers and facilitators to breastfeeding in the context in which an incentive intervention is implemented. However there is the potential for “reverse innovation” in public health [46, 47], where interventions designed for a low-income context are adapted and implemented in a high-income context. Therefore for this topic, where the number of studies is small, bringing together evidence from across settings may be useful for public health organisations exploring this.

## Conclusion

There is some evidence that incentives may improve breastfeeding outcomes. However, the small number of published studies with heterogenous interventions, samples, and outcomes, mean that it is not possible to draw firm conclusions about their overall or relative effectiveness. Future research on incentives for breastfeeding should be evaluated in appropriately powered studies, include cost-effectiveness analysis, and more broadly, researchers in this area should work towards an agreed set of breastfeeding outcomes to improve comparability and future synthesis of evidence.

## Supplementary Information

Supplementary Material 1.

Supplementary Material 2.

## Data Availability

Search strategies are available in the supplementary material. There is no unpublished data involved in the research described here.
