# Supplementary material for: Evidence for incentive-based strategies to promote breastfeeding: a systematic literature review of randomised controlled trials
Source: BMC Pregnancy Childbirth. 2025 Nov 19;25:1246. doi: 10.1186/s12884-025-08405-2 (PMC12628927; doi:10.1186/s12884-025-08405-2)
Supplement: Supplementary file 1 — Supplementary Material 1. [file 12884_2025_8405_MOESM1_ESM.docx]

**Supplementary material**

**Evidence for incentive-based strategies to promote breastfeeding: a systematic literature review of randomised controlled trials**

**Table S1**. Search strategies

| MEDLINE and Embase | (Breastfeeding OR breast feeding OR Breastfed OR breast fed OR infant feeding OR human milk OR breastmilk OR breast milk OR lactation)  AND  (award OR gift OR money OR tax credit OR cash OR entice* OR incentiv* OR payment OR prize OR reward)  AND  (randomi* OR RCT)  NOT  (protocol)  Search titles and abstracts  Limit 2000-31/08/2024  Limit humans  Limit English language |
| --- | --- |
| Scopus | TITLE-ABS ((breastfeeding OR "breast feeding" OR breastfed OR "breast fed" OR "infant feeding" OR "human milk" OR breastmilk OR "breast milk" OR lactation))  AND  TITLE-ABS ((award OR gift OR money OR "tax credit" OR cash OR entice* OR incentiv* OR payment OR prize OR reward))  AND  TITLE-ABS ((randomi* OR rct ) )  AND NOT  TITLE ((protocol))  AND  PUBYEAR > 1999 AND PUBYEAR < 2025  AND  (LIMIT-TO (LANGUAGE, "English”))  AND  (LIMIT-TO (EXACTKEYWORD, "Human" ) OR LIMIT-TO ( EXACTKEYWORD , "Humans" ) ) |
| CINAHL and PsychInfo | AB ((Breastfeeding OR breast feeding OR Breastfed OR breast fed OR infant feeding OR human milk OR breastmilk OR breast milk OR lactation))  AND  AB ((award OR gift OR money OR tax credit OR cash OR entice* OR incentiv* OR payment OR prize OR reward))  AND  AB ((randomi* OR RCT))  NOT  TI((protocol))  Limit 01/2000-08/2024  Limit humans  Limit English language |

**Table S2.** Studies which were excluded following full text review

| **First author (year of publication)** | **Title** | **Reason for exclusion** |
| --- | --- | --- |
| Grijalva-Eternod (2023) (1) | Evaluation of conditional cash transfers and mHealth audio messaging in reduction of risk factors for childhood malnutrition in internally displaced persons camps in Somalia: A 2 x 2 factorial cluster-randomised controlled trial. | The intervention was a conditional cash transfer, and the comparator was an unconditional cash transfer, so it is not incentive vs. no incentive |
| Wambach (2011) (2) | A Randomized Controlled Trial of Breastfeeding Support and Education for Adolescent Mothers | All breastfeeding participants were eligible for financial incentives, not just the experimental group participants. |

**REFERENCES**

1. Grijalva-Eternod CS, Jelle M, Mohamed H, Waller K, Hussein BO, Barasa E, et al. Evaluation of conditional cash transfers and mHealth audio messaging in reduction of risk factors for childhood malnutrition in internally displaced persons camps in Somalia: A 2 × 2 factorial cluster-randomised controlled trial. PLoS Med. 2023;20(2).

2. Wambach KA, Aaronson L, Breedlove G, Domian EW, Rojjanasrirat W, Yeh HW. A Randomized Controlled Trial of Breastfeeding Support and Education for Adolescent Mothers. West J Nurs Res. 2011 Jun 1;33(4):486–505.

3. Critical Appraisal Skills Programme. CASP Randomised Controlled Trial Checklist [Internet]. 2021. Available from: https://casp-uk.net/casp-tools-checklists/randomised-controlled-trial-rct-checklist/

4. Relton C., Strong M., Thomas K.J., Whelan B., Walters S.J., Burrows J., et al. Effect of financial incentives on breastfeeding a cluster randomized clinical trial. JAMA Pediatr. 2018;172(2):Y.

5. Anokye N, Coyle K, Relton C, Walters S, Strong M, Fox-Rushby J. Cost-effectiveness of offering an area-level financial incentive on breast feeding: a within-cluster randomised controlled trial analysis. Arch Dis Child. 2020;105(2):155–9.

6. Rossouw L., Burger R.P., Burger R. Testing an Incentive-Based and Community Health Worker Package Intervention to Improve Maternal Health and Nutrition Outcomes: A Pilot Randomized Controlled Trial. Matern Child Health J. 2021;25(12):1913–22.

7. Kurdi S., Figueroa J.L., Ibrahim H. Nutritional training in a humanitarian context: Evidence from a cluster randomized trial. Matern Child Nutr. 2020;16(3):e12973.

8. Washio Y., Humphreys M., Colchado E., Sierra-Ortiz M., Zhang Z., Collins B.N., et al. Incentive-based intervention to maintain breastfeeding among low-income Puerto Rican mothers. Pediatrics. 2017;139(3):e20163119.

9. Kandpal E., Alderman H., Friedman J., Filmer D., Onishi J., Avalos J. A conditional cash transfer program in the philippines reduces severe stunting. J Nutr. 2016;146(9):1793–800.

10. Finch C, Daniel EL. Breastfeeding education program with incentives increases exclusive breastfeeding among urban WIC participants.(Research And Professional Briefs). Journal of the American Dietetic Association. 2002;102(7):981–5.
